# Headache and Alexithymia in Children and Adolescents: What Is the Connection?

**DOI:** 10.3389/fpsyg.2018.00048

**Published:** 2018-02-01

**Authors:** Giulia Natalucci, Noemi Faedda, Dario Calderoni, Rita Cerutti, Paola Verdecchia, Vincenzo Guidetti

**Affiliations:** ^1^Department of Paediatric and Child and Adolescent Neuropsychiatry, “Sapienza” University of Rome, Rome, Italy; ^2^Behavioural Neuroscience, Department of Paediatric and Child and Adolescent Neuropsychiatry, “Sapienza” University of Rome, Rome, Italy; ^3^Department of Dynamic and Clinical Psychology, Sapienza University of Rome, Rome, Italy

**Keywords:** headache, migraine, tension type headache, alexithymia, children, adolescents

## Abstract

**Background:** Headache is one of the most common complaints in children and adolescents and comorbidity rates are very high and the major associated diseases are depression, anxiety, atopic disorders, sleep, and behavioral disorders. In recent years, it has been highlighted that difficulties regulating emotions such as alexithymia have also been associated with diagnosis of somatization.

**Methods:** We carried out a mini review analyzing the relation between alexithymia and primary headache (e.g., migraine and tension type headache) in children and adolescents by synthesizing the relevant studies in the literature on PubMed, PsycINFO, and Google Scholar. Search terms were “alexithymia” combined with the “primary headache,” “migraine,” “tension type headache,” “children,” and “adolescents.”

**Results:** All analyzed studies found higher levels of alexithymia in children and adolescents with headache than control groups but there are different opinions about the relationship between headache and alexithymia. For example, some studies suggest that the association between headache and alexithymia in children may be due to an incomplete development of emotive competency or a general immature cognitive development, instead other studies found a correlation between headache symptoms, insecure attachment, and alexithymia. There seems to be also differences between children with migraine compared to those with tension type headache (TTH).

**Conclusion:** There are some studies on adults suffering from headache or migraine and alexithymia, but there is only a moderate amount of research on pediatric age with different opinions and theories about this relationship. Further studies on children and adolescents are necessary to effectively understand this relationship and to help children to reduce headache and improve emotional consciousness.

## Introduction on primary headache

Primary headaches are the most frequent complaints among the pediatric population with migraine and tension type headache (TTH) being the most common type. This disorder causes individual suffering and impairments in quality of life, daily activities (Dyb et al., 2015), school attendance (Rousseau-Salvador et al., 2014) and a variety of serious family issues (Wöber-Bingöl, 2013). Moreover, primary headache can be associated with several comorbid conditions which may worsen headache symptomatology, prognosis, therapeutic selection and post- therapeutic outcomes (Mark, 2015). In the pediatric population, tension type headache, and migraine are commonly associated with various diseases. For instance, psychiatric and neurological comorbidity, in particular sleep disorders, anxiety and depression, epilepsy and ADHD (Bellini et al., 2013). It has also been shown an association with medical disorders such as atopy, cardiovascular disease, especially ischemic stroke and Patent Foramen Ovale (PFO) (Bellini et al., 2013). In some studies, it has been shown that children with migraine had significantly higher levels of internalizing and somatic symptoms, as well as social and family problems relative to those without headache and had higher levels of somatic symptoms than children with tension-type headache (Anttila et al., 2004; Arruda and Bigal, 2012). In recent years, alexithymia has been studied among the emotional disturbances associated with somatic symptoms including primary headache, and there are some different opinions about this relationship.

## Alexithymia construct

Alexithymia has also been recognized to be associated with several somatic illnesses and mental disorders. The term alexithymia was coined by Sifneos in 1973 (Sifneos, 1973) (from the Greek; a = lack, lexis = word, thymos emotion), concerning a cognitive-affective disturbance that affects the way individuals experience and express their emotions, internal states and feelings (Taylor, 1984). There is a reduced ability to identify and describe feelings, a limited imagination, and a concrete, externally- oriented way of thinking. Recently, it has been proposed a two-dimensional alexithymia. The affective dimension refers to the level of subjective emotional experience and comprises of a reduced ability to experience emotional feelings (emotionalizing factor), and diminished imaginative capabilities (fantasizing factor) (Vorst and Bermond, 2001; Koelen et al., 2015). The cognitive dimension refers to the more verbal-explicit aspects, such as the inability to verbalize emotions and to differentiate between emotions, as well as between emotions and somatic sensations and the presence of an “externally oriented thinking” style (Bermond et al., 2010; Goerlich-Dobre et al., 2014). Many studies have highlighted the association between alexithymic traits and psychiatric diseases. Most of them have found a positive association with internalizing disorders such as depression and anxiety traits (Leweke et al., 2012; Scimeca et al., 2014; Li et al., 2015). Additionally, alexithymia is co-present with eating (Lulé et al., 2014), behavioral disorders (e.g., severe disruptive behavior) (Manninen et al., 2011) and personality disorders such as borderline personality (Loas et al., 2012). Other studies focused on the association between alexithymia and mother's little or lack of education and broken family, disadvantageous living conditions in childhood (Joukamaa et al., 2007) and insecure attachment strategies (Besharat and Khajavi, 2013; Koelen et al., 2015). Finally, some studies have noticed that children with recurrent pain disorders (i.e., migraine, tension type headache, skeletal muscle pain, abdominal pain) compared to healthy controls, show higher level of alexithymic features (Allen et al., 2011; Cerutti et al., 2016). For example, in one study conducted by Burba et al. (2006), adolescents with somatoform disorder had higher levels of alexithymia and anxiety than healthy adolescent control subjects.

## Relationship between alexithymia and primary headache in children and adolescents: what we know and what we can hypothesize?

There are a few studies devoted to the investigation of the possible link between headache and alexithymia in adults (De Andrade et al., 2013), and even fewer on children and adolescent populations. For example, it has been observed that adults with migraine or TTH had a higher alexithymia level, compared to healthy controls (Wise et al., 1994). Furthermore, there would seem to be no difference between the severity of alexithymia among the cases of experiencing episodic vs. chronic TTH (Yücel et al., 2002). The association between alexithymia and primary headache in children and adolescence is not well-known yet. Some authors have tried to study the phenomenon and provide some hypotheses. In a pilot study conducted by Gatta et al. (2011), the aim was to analyse alexithymic features in children with primary headache (both TTH and migraine). They found a significant association between TTH and alexithymia in the experimental group. Children with TTH had major alexithymic problems compared to children with migraine and the control group. Two dimensions were particularly compromised in children with headache: recognizing their own feelings and tendency for operatory thought. On account of these results, the Authors supposed alexithymia to create a condition in which feelings or emotions (emotive or somatic), when not distinguished, may undergo a process of reinforcement and become a symptom of disease. Moreover, there were some children with high level of alexithymia in their control group and the authors suggested that they may still be in a developmental phase also regarding emotional recognition and awareness, in accordance with the Lane and Schwartz (Lane and Schwartz, 1987) cognitive-developmental model of emotions. In a more recent study, Gatta et al. (2015) investigated alexithymic features in children and adolescents with primary headache (both migraine and tension type headache) and in their mothers, in order to examine the possibility of a relationship between headache and emotional regulation, in particular alexithymia. They have confirmed their prior results: a significant relation between TTH and alexithymia was found, but not between Migraine and alexithymia. They assumed that the differences between TTH and migraine may be due to different pathogenesis of the two disorders. Genetic factors may underline the etiology of migraine more than TTH, while TTH has a more complex multifactorial pathogenesis, influenced also by the familial and social environment. In fact, primary headache is more common in families with a history of psychological disorders (Galli et al., 2009). Lastly, their results partially support the hypothesis that alexithymia in a child may correspond to a similar kind of emotional dysregulation in the mother, emphasizing a connection between these two factors (Jørgensen et al., 2007). Even if they have not found significant alexithymic differences between mothers of the three groups (Migraine, TTH and control), they believed that emotional difficulties may lie in other aspects of emotional regulation implied in mood and anxiety disorders, which have a high prevalence in parents of children with headache (Feldman et al., 2010). In line with this study, Cerutti et al. (2016) examined the association between migraine and alexithymia, exploring the hypothesis of alexithymia predicting psychopathological symptoms in adolescents and mothers with migraines. Furthermore, determining any differences between adolescents and their mothers suffering from migraine vs. adolescents and mothers in the control groups (and the relation with both mother and child with migraine). In contrast with Gatta's results, they discovered that adolescents and their mothers with migraine had higher rates of alexithymia. Additionally, they demonstrated that both adolescents and mothers, both suffering from migraine, also appear to experience greater psychological distress than the adolescents and mothers in the control groups. In light of these results, they supposed a possible intergenerational transmission of alexithymia and a child's alexithymia may reflect the mother's deficit in regulating emotions and expressing feelings (Yürümez et al., 2014). At this point, it is fundamental to analyse the relationship between headache, attachment style and alexithymia. It has been observed a connection between migraine features (high attack frequency and severe pain intensity) and attachment style, especially the ambivalent one (Tarantino et al., 2017). Moreover, children with migraine and with ambivalent attachment style report higher symptoms of anxiety, depression, and somatization. Also, Williams et al (Williams et al., 2017) showed that the somatic condition of migraine acted as a stressor, reducing children's perception of the security of their parental attachments. It seems that children with migraine exhibit a significantly higher prevalence of avoidant attachment and a lower prevalence of secure attachment (Esposito et al., 2013). From these evidence, a potential influence of attachment style on emotional expression and primary headache may be taken into consideration. Some genetic studies have attempted to understand whether alexithymia is a transmissible construct. For example, Baughman et al., have found different results in two separate studies. In the first one (Baughman et al., 2011), the phenotypic correlations between alexithymia and emotional intelligence traits seemed to be attributable to correlated genetic and correlated non-shared environmental factors. In the second one (Baughman et al., 2013), they found that, in addition to the antecedent factors, there was also an influence by shared-environment. The genetic component observed supports previous suggestions that biological base may contribute to the development of alexithymia (Larsen et al., 2003). However, Picardi et al. (2011) suggested that emotional distress may confound the estimation of the heritability of alexithymia, corroborating the notion that alexithymia is substantially heritable and enhancing the role of unshared environmental factors.

Cerebral anomalies may be underlying both alexithymia and migraine, but there is still no certainty and accuracy about this correlation. In a research conducted by Liu et al. (2013) gray matter and white matter changes have been compared in a group of the early clinical stage of migraine patients without aura for 1 year. When they re-examined the patients, a year following the first observation, gray matter reduction was observed in the dorsolateral and medial part of the superior frontal gyrus, orbitofrontal cortex, hippocampus, precuneus, and primary and secondary somatosensory cortices. However, no differences in white matter changes were found. Thus, a possible connection between migraine and alexithymia may be due to changes in specific cerebral areas. In fact, altered gray matter volume (Kano and Fukudo, 2013; Grabe et al., 2014) and a reduced activation in the posterior cingulate cortex (Mantani et al., 2005) seem to be related to lower alexithymia levels.

## Conclusions

Mechanisms through which psychological mechanisms influence a physical disorder are complex and difficult to detect with the available tools (Kano and Fukudo, 2013). Studies on children and adolescents are often not simple and with *bias*. Indeed, the majority of studies involve children and adolescents together (aged between 8 and 15 years), and since childhood and adolescence are ages of change and the psychological characteristics are constantly developing, some children might find it difficult to verbally express themselves. This might depend on their incomplete emotional development and immature brain areas (i.e., 28). Moreover, some bias can influence the assessment of alexithymia. Alexithymia Questionnaire for Children is the only tool for measuring the alexithymic construct at a developmental age, but it has some critical issues (Rosenberg et al., 2016). The major problem is the validity of self- report measures of alexithymia. Especially in childhood there are several problems with self-report measurement due to problems in understanding questions, reading difficulties, social desirability, and last but not least, the lack of a general understanding of their internal states. Expressing emotions, feelings and using imagination is moresimple for an adult in respect to a child, on account of their experience and acquired competences (both verbal and emotional) (Lumley et al., 2005). The concept that the headache and alexithymia in children are connected is supported by the literature, however, this is rather limited. Further studies on children and adolescents and more accurate instruments are necessary to better understand this relationship and to help children in reducing headache and improve emotional consciousness. Research psychologists and physicians have to work together to improve the existent questionnaire and knowledge about the origins of both alexithymia and primary headache in order to reduce severity of headache and psychological suffering of children and adolescents.

## Author contributions

GN, NF, and VG conceived and designed the study. DC, RC, PV, and VG were responsible for critical revision of this manuscript. All authors approved the final version of this manuscript.

### Conflict of interest statement

The authors declare that the research was conducted in the absence of any commercial or financial relationships that could be construed as a potential conflict of interest.

## References

[B1] Allen L. B. Lu Q. Tsao J. C. I. Hayes L. P. Zeltzer L. K. (2011). Depression partially mediates the relationship between alexithymia and somatization in a sample of healthy children. J. Health Psychol. 16, 1177–1186. 10.1177/1359105311402407 21464112 PMC3132307

[B2] Anttila P. Sourander A. Metsähonkala L. Aromaa M. Helenius H. Sillanpää M. (2004). Psychiatric symptoms in children with primary headache. J. Am. Acad. Child. Adolesc. Psychiatry 43, 412–419. 10.1097/00004583-200404000-00007 15187801

[B3] Arruda M. A. Bigal M. E. (2012). Behavioral and emotional symptoms and primary headaches in children: a population-based study. Cephalalgia 32, 1093–1100. 10.1177/0333102412454226 22988005

[B4] Baughman H. M. Schermer J. A. Veselka L. Harris J. Vernon P. A. (2013). A behavior genetic analysis of trait emotional intelligence and alexithymia: a replication. Twin Res. Hum. Genet. 16, 554–559. 10.1017/thg.2012.151 23298794

[B5] Baughman H. M. Schwartz S. Schermer J. A. Veselka L. Petrides K. V. Vernon P. A. (2011). A behavioral-genetic study of alexithymia and its relationships with trait emotional intelligence. Twin Res. Hum. Genet. 14, 539–543. 10.1375/twin.14.6.539 22506309

[B6] Bellini B. Arruda M. Cescut A. Saulle C. Persico A. Carotenuto M.. (2013). Headache and comorbidity in children and adolescents. J. Headache Pain. 14:79. 10.1186/1129-2377-14-79 24063537 PMC3849985

[B7] Bermond B. Bierman D. J. Cladder M. A. Moormann P. P. Vorst H. C. (2010). The cognitive and affective alexithymia dimensions in the regulation of sympathetic responses. Int. J. Psychophysiol. 75, 227–233. 10.1016/j.ijpsycho.2009.11.004 19951721

[B8] Besharat M. A. Khajavi Z. (2013). The relationship between attachment styles and alexithymia: mediating role of defense mechanisms. Asian J. Psychiatry 6, 571–576. 10.1016/j.ajp.2013.09.003 24309875

[B9] Burba B. Oswald R. Grigaliunien V. Neverauskiene S. Jankuviene O. Chue P. (2006). A controlled study of alexithymia in adolescent patients with persistent somatoform pain disorder. Can. J. Psychiatry 51, 468–471. 10.1177/070674370605100709 16838829

[B10] Cerutti R. Valastro C. Tarantino S. Valeriani M. Faedda N. Spensieri V.. (2016). Alexithymia and psychopathological symptoms in adolescent outpatients and mothers suffering from migraines: a case control study. J. Headache Pain 17, 39. 10.1186/s10194-016-0640-y 27093870 PMC4837193

[B11] De Andrade R. V. Vieira D. C. Gomes W. B. Gauer G. (2013). Alexithymia and its impact on quality of life in a group of Brazilian women with migraine with aura. J. Headache Pain 14, 17–27 10.1186/1129-2377-14-18 23565860 PMC3620425

[B12] Dyb G. Stensland S. Zwart J.A. (2015). Psychiatric comorbidity in childhood and adolescence headache. Curr. Pain Headache Rep. 19, 5. 10.1007/s11916-015-0479-y 25754599 PMC4353875

[B13] Esposito M. Parisi L. Gallai B. Marotta R. Di Dona A. Lavano S. M.. (2013). Attachment styles in children affected by migraine without aura. Neuropsychiatr. Dis. Treat. 9, 1513–1519. 10.2147/NDT.S52716 24124370 PMC3794987

[B14] Feldman J. M. Ortega A. N. Koinis-Mitchell D. Kuo A. A. Canino G. (2010). Child and family psychiatric and psychological factors associated with child physical health problems: results from the Boricua youth study. J. Nerv. Ment. Dis. 198, 272–279. 10.1097/NMD.0b013e3181d61271 20386256 PMC2958697

[B15] Galli F. Canzano L. Scalisi T. G. Guidetti V. (2009). Psychiatric disorders and headache familial recurrence: a study on 200 children and their parents. J. Headache Pain 10, 187–197. 10.1007/s10194-009-0105-7 19352592 PMC3451992

[B16] Gatta M. Canetta E. Zordan M. Spoto A. Ferruzza E. Manco I.. (2011). Alexithymia in juvenile primary headache sufferers: a pilot study. J. Headache Pain 12, 71–80. 10.1007/s10194-010-0248-6 20730593 PMC3072508

[B17] Gatta M. Spitaleri C. Balottin U. Spoto A. Balottin L. Mangano S.. (2015). Alexithymic characteristics in pediatric patients with primary headache: a comparison between migraine and tension-type headache. J. Headache Pain 16, 98. 10.1186/s10194-015-0572-y 26607363 PMC4659793

[B18] Goerlich-Dobre K. S. Bruce L. Martens S. Aleman A. Hooker C. I. (2014). Distinct associations of insula and cingulate volume with the cognitive and affective dimensions of alexithymia. Neuropsychologia. 53, 284–292. 10.1016/j.neuropsychologia.2013.12.006 24334109

[B19] Grabe H. J. Wittfeld K. Hegenscheid K. Hosten N. Lotze M. Janowitz D.. (2014). Alexithymia and brain gray matter volumes in a general population sample. Hum. Brain Mapp. 35, 5932–5945. 10.1002/hbm.22595 25081815 PMC6869686

[B20] Jørgensen M. M. Zachariae R. Skytthe A. Kyvik K. (2007). Genetic and environmental factors in alexithymia: a population-based study of 8785 Danish twin pairs. Psychother. Psychosom. 76, 369–375 10.1159/000107565 17917473

[B21] Joukamaa M. Taanila A. Miettunen J. Karvonen J. T. Koskinen M. Veijola J. (2007). Epidemiology of alexithymia among adolescents. J. Psychosom. Res. 63, 373–376. 10.1016/j.jpsychores.2007.01.018 17905044

[B22] Kano M. Fukudo S. (2013). The alexithymic brain: the neural pathways linking alexithymia to physical disorders. Biopsychosoc. Med. 7:1. 10.1186/1751-0759-7-1 23302233 PMC3563604

[B23] Koelen J. A. Eurelings-Bontekoe E. H. Stuke F. (2015). Luyten PInsecure attachment strategies are associated with cognitive alexithymia in patients with severe somatoform disorder. Int. J. Psychiatry Med. 49, 264–278. 10.1177/0091217415589303 26060261

[B24] Lane R. D. Schwartz G. E. (1987). Levels of emotional awareness: a cognitive-developmental theory and its application to psychopathology. Am. J. Psychiatry 144, 133–143. 3812780 10.1176/ajp.144.2.133

[B25] Larsen J. K. Brand N. Bermond B. Hijman R. (2003). Cognitive and emotional characteristics of alexithymia: a review of neurobiological studies. J. Psychosom. Res. 54, 533–541. 10.1016/S0022-3999(02)00466-X 12781307

[B26] Leweke F. Leichsenring F. Kruse J. Hermes S. (2012). Is alexithymia associated with specific mental disorders? Psychopathology 45, 22–28. 10.1159/000325170 22123513

[B27] Li S. Zhang B. Guo Y. Zhang J. (2015). The association between alexithymia as assessed by the 20- item toronto alexithymia scale and depression: a meta-analysis. Psychiatry Res. 227, 1–9. 10.1016/j.psychres.2015.02.006 25769520

[B28] Liu J. Lan L. Li G. Yan X. Nan J. Xiong S.. (2013). Migraine-related gray matter and white matter changes at a 1-year follow-up evaluation. J. Pain 14, 1703–1708. 10.1016/j.jpain.2013.08.013 24290450

[B29] Loas G. Speranza M. Pham-Scottez A. Perez-Diaz F. Corcos M. (2012). Alexithymia in adolescents with borderline personality disorder. J. Psychosom. Res. 72, 147–152. 10.1016/j.jpsychores.2011.11.006 22281457

[B30] Lulé D. Schulze U. M. Bauer K. Schöll F. Müller S. Fladung A. K.. (2014). Anorexia nervosa and its relation to depression, anxiety, alexithymia and emotional processing deficits. Eat. Weight Disord. 19, 209–216. 10.1007/s40519-014-0101-z 24474662

[B31] Lumley M. A. Gustavson B. J. Partridge R. T. Labouvie-Vief G. (2005). Assessing alexithymia and related emotional ability constructs using multiple methods: interrelationships among measures. Emotion 5, 329–342. 10.1037/1528-3542.5.3.329 16187868

[B32] Wise T. N. Mann L. S. Jani N. Jani S. (1994). Illness beliefs and alexithymia in headache patients. Headache. 34, 362–365. 10.1111/j.1526-4610.1994.hed3406362.x 7928316

[B33] Manninen M. Therman S. Suvisaari J. Ebeling H. Moilanen I. Huttunen M.. (2011). Alexithymia is common among adolescents with severe disruptive behavior. J. Nerv. Ment. Dis. 199, 506–509. 10.1097/NMD.0b013e3182214281 21716065

[B34] Mantani T. Okamoto Y. Shirao N. Okada G. Yamawaki S. (2005). Reduced activation of posterior cingulate cortex during imagery in subjects with high degrees of alexithymia: a functional magnetic resonance imaging study. Biol. Psychiatry 57, 982–990. 10.1016/j.biopsych.2005.01.047 15860338

[B35] Mark W. (2015). Weatherall The diagnosis and treatment of chronic migraine. Ther. Adv. Chronic. Dis. 6, 115–123. 10.1177/2040622315579627 25954496 PMC4416971

[B36] Picardi A. Fagnani C. Gigantesco A. Toccaceli V. Lega I. Stazi M. A. (2011). Genetic influences on alexithymia and their relationship with depressive symptoms. J. Psychosom. Res. 71, 256–263. 10.1016/j.jpsychores.2011.02.016 21911104

[B37] Rosenberg N. Rufer M. Lichev V. Ihme K. Grabe H. J. Kugel H. (2016). Observer-rated alexithymia and its relationship with the five-factor-model of personality. Psychol. Belg. 56, 118–134. 10.5334/pb.302 PMC5854197 30479433

[B38] Rousseau-Salvador C. Amouroux R. Annequin D. Salvador A. Tourniaire B. Rusinek S. (2014). Anxiety, depression and school absenteeism in youth with chronic or episodic headache. Pain Res. Manag. 19, 235–240. 10.1155/2014/541618 24911174 PMC4197750

[B39] Scimeca G. Bruno A. Cava L. Pandolfo G. Muscatello M. R. Zoccali R. (2014). The relationship between alexithymia, anxiety, depression, and internet addiction severity in a sample of Italian high school students. Sci. World J. 2014:504376. 10.1155/2014/504376 25401143 PMC4221883

[B40] Sifneos P. E. (1973). The prevalence of alexithymic characteristics in psychosomatic patients. Psychother. Psychosom. 22, 255–262 10.1159/000286529 4770536

[B41] Tarantino S. De Ranieri C. Dionisi C. Gagliardi V. Paniccia M. F. Capuano A.. (2017). Role of the attachment style in determining the association between headache features and psychological symptoms in migraine children and adolescents. an analytical observational case-control study. Headache 57, 266–275. 10.1111/head.13007 28058729

[B42] Taylor G. J. (1984). Alexithymia: concept, measurement, and implications for treatment. Am. J. Psychiatry. 141, 725–732. 6375397 10.1176/ajp.141.6.725

[B43] Vorst H. Bermond B. (2001). Validity and reliability of the bermond-vorst alexithymia questionnaire. Pers. Indiv. Differ. 30, 413–434 10.1016/S0191-8869(00)00033-7

[B44] Williams R. Leone L. Faedda N. Natalucci G. Bellini B. Salvi E.. (2017). The role of attachment insecurity in the emergence of anxiety symptoms in children and adolescents with migraine: an empirical study. J. Headache Pain 18, 62. 10.1186/s10194-017-0769-3 28560542 PMC5449355

[B45] Wöber-Bingöl C. (2013). Epidemiology of migraine and headache in children and adolescents. Curr. Pain Headache Rep. 17:341. 10.1007/s11916-013-0341-z 23700075

[B46] Yücel B. Kora K. Ozyalçín S. Alçalar N. Ozdemir O. Yücel A.. (2002). Depression, automatic thoughts, alexithymia, and assertiveness in patients with tension-type headache. Headache. 42, 194–199 10.1046/j.1526-4610.2002.02051.x 11903542

[B47] Yürümez E. Akça Ö. F. Ugur Ç. Uslu R. I. Kiliç B. G. (2014). Mothers' alexithymia, depression and anxiety levels and their association with the quality of mother-infant relationship: a preliminary study. Int. J. Psychiatry Clin. Pract. 18, 190–196. 10.3109/13651501.2014.940055 24994481

